# Comparative analysis of repeat content in plant genomes, large and small

**DOI:** 10.3389/fpls.2023.1103035

**Published:** 2023-07-14

**Authors:** Joris Argentin, Dan Bolser, Paul J. Kersey, Paul Flicek

**Affiliations:** ^1^ Institut de Biologie en Santé, Centre Hospitalier Universitaire (CHU) d’Angers, Angers, France; ^2^ European Molecular Biology Laboratory, European Bioinformatics Institute, Cambridge, United Kingdom; ^3^ Digital Revolution, Royal Botanic Gardens, Kew, Richmond, United Kingdom

**Keywords:** transposons, plants, pipeline, annotation, comparative analysis

## Abstract

The DNA Features pipeline is the analysis pipeline at EMBL-EBI that annotates repeat elements, including transposable elements. With Ensembl’s goal to stay at the cutting edge of genome annotation, we proved that this pipeline needed an update. We then created a new analysis that allowed the Ensembl database to store the repeat classification from the PGSB repeat classification (Recat). This new dataset was then fetched using Perl scripts and used to prove that the pipeline modification induced a gain in sensitivity. Finally, we performed a comparative analysis of transposable element distribution in all plant species available, raising new questions about transposable elements in certain branches of the taxonomic tree.

## 1 Introduction

Transposable elements (TEs) are a major group of genomic repetitive elements. TEs encompass many genomic structures that all have in common the ability to move from one genomic location to another in a process called transposition. Transposition itself covers various mechanisms.

Approximately 3% to 80% of a plant’s genome is composed of TEs. By their nature as repetitive sequences, TEs are major contributors, with whole genome duplications, to the large genome size reported in plant genomes (Muotri et al., 2007). The predominance of transposons makes repeat content detection essential. Each species has its own history of repeat expansions and removals, which poses intriguing questions about evolution, host control, transposon countermeasures, and other factors that influence genomic repeats.

### 1.1 Transposition mechanisms

There are two main ways for a repeat element to move in the genome. These two ways to perform a transposition will define the main classes of repeat elements.

The replicative transposition, or retrotransposition, implies a reverse transcription of the TEs. LTR-Retrotransposons are flanked by long terminal repeat (LTR) and code for their own transposition proteins. As for the non-LTR coding elements, long interspersed nuclear elements (LINEs) also code for their own transposition proteins, while short interspersed nuclear elements (SINEs) are non-autonomous. Both LTR and non-LTR transpositions are considered a “copy–paste system” and result in the duplication of the repeat element.

The other mechanism, similar to a “cut–paste” system, is called conservative transposition. It involves the transposase, coded by the gene in the transposon sequence, and inverted tandem repeats (ITRs). The transposase will then bind to both ITRs, cleave the DNA, forming a circular structure, and transport the TEs to the target site.

### 1.2 Classifications of transposable elements

TEs are not under large selection pressure, so multiple copies accumulate mutations, to the point of losing all transposition activities. This accumulation of mutations can also play beneficial roles in evolutionary processes (Chénais et al., 2012), creating variety in genetic portions that can be transferred with TEs. Therefore, transposable elements have a relatively short transposition activity, and active elements in modern genomes are rare. This degeneration can also happen with repeat elements getting inserted within other elements, ultimately leading to complex, nested, and degenerated structures, making homology-defined families not straightforward.

Wicker et al. (2007) defined a transposable element family with these criteria: “two elements belong to the same family if they share 80% (or more) sequence identity in at least 80% of their coding or internal domain, or within their terminal repeat regions, or in both”.

### 1.3 TE detection and annotation

During gene annotation processes, repeat elements are masked to minimize unwanted transposon-related gene calls due to the repetitive nature of transposable elements. This detection is mainly performed by searching the genome sequence against a reference library, like RepeatMasker^1^. libraries are automatically built from motif discovery tools based on repetitiveness (Benson, 1999), specific TE structures, or comparative genomics (Ou et al., 2019). However, these automated methods have flaws in accuracy and still need manual annotation. EMBL-EBI, displaying annotation information for scientists worldwide in the Ensembl browser, must be on the cutting edge of transposable element annotation. In 2020, repeat elements at EBI were annotated by the DNA Features pipeline. This pipeline ran RepeatMasker with the Repbase (Bao et al., 2015) repeat library.

In the current work, we aimed to extend our existing pipeline for repeat annotation to produce a comprehensive catalog of repeat families across the complete range of sequenced plant genomes. We ran the existing repeat annotation pipeline across all genomes in Ensembl Plants and compared the results to the literature. This was necessary to assess the need to implement a new, more specific repeat library. We had to extend the pipeline to apply and import repeat classification from the PGSB repeat classification (REcat; Nussbaumer et al., 2013), similar to the way repeat classification is added from Repbase. The new data generated using the REcat were used to quantify the improvement in TE detection. REcat integrates existing classifications for repetitive elements into a more detailed hierarchical tree structure. The resulting catalog of classified repeats was then compared across the taxonomic space to establish the evolutionary trends of repeat expansion and to extend understanding of chromosomal architecture in plants.

## 2 Materials and methods

### 2.1 Literature monitoring

#### 2.1.1 Bibliographic research

The repeat distribution table, for the barley (*Hordeum vulgare*) genome described in Mascher et al. (2017), was used as the working base in our repeat statistics spreadsheet. For its completeness, this table was also used as the standard of quality for other repeat distribution tables. To find genome-wide repeat distribution reports, two queries on PubMed were made: one using Mesh terms (*((Genus + species name[All Fields]) AND Interspersed Repetitive Sequences[MeSH Terms]) AND plants, genetics[MeSH Terms]*) and the other for repeat distribution tables in genome-wide assembly reports *via* the linked articles in the National Center for Biotechnology Information (NCBI) Genome website (*Genus species[orgn]*).

#### 2.1.2 Quality control of repeat distribution tables

All repeat distribution tables found using both of the methods described in the previous paragraph had to have a quality at least equivalent to the previous standard. A table could still pass quality control if in a repeat type a superfamily-related row was missing, but all other family rows for this type were present. In that particular case, statistics for the missing row were considered zero. The various classifications used in the articles were normalized using the PGSB repeat classification (Nussbaumer et al., 2013). Due to the quality control, processing of annotation statistics was only performed on eight of the 53 species (including a cultivar) present in the database: two genome-wide repeat distribution studies were found for *Brachypodium distachyon* (Initiative, 2010) and *Amborella trichopoda* (Zuccolo et al., 2011); four assembly reports that comprised relevant repeat annotation statistics were found for Japanese and Indian rice (Mahesh et al., 2016) (*Oryza sativa* sp. *japonica* cv. *Nipponbare* and sp. *indica* cv. *93-11*), soybean (Schmutz et al., 2010) (*Glycine max*), cacao (Motamayor et al., 2013) (*Theobroma cacao*), and maize (Jiao et al., 2017) (*Zea mays*) respectively.

### 2.2 Comparison of repeat distributions between the DNA Features pipeline and scientific articles

The statistics were stored in a Google Sheets spreadsheet (Bolser et al., 2015). This spreadsheet comprised six metrics (percentage of the genome covered, percentage of total transposable element length, base pairs covered, number of features, size in Mbp, and average length in bp) for classes, superfamilies, and the main families of transposable elements, similar to the statistics presented in Mascher et al. (2017). Repeat distribution statistics from the literature were also stored in this spreadsheet, next to their corresponding distribution from the pipeline. The percentage of the genome covered and the number of features for all transposable elements (or the “Transposable elements” repeat sequence group) were used as metrics to compare annotation performances between the initial and modified DNA Features pipeline and the literature used as reference.

### 2.3 Statistics and software

#### 2.3.1 Cluster computing

Data processing of the pipeline was performed on the EBI cluster monitored by the LSF^2^ and eHive^3^ systems.

#### 2.3.2 Annotation of mobile elements in the pipeline

**Figure 1:**
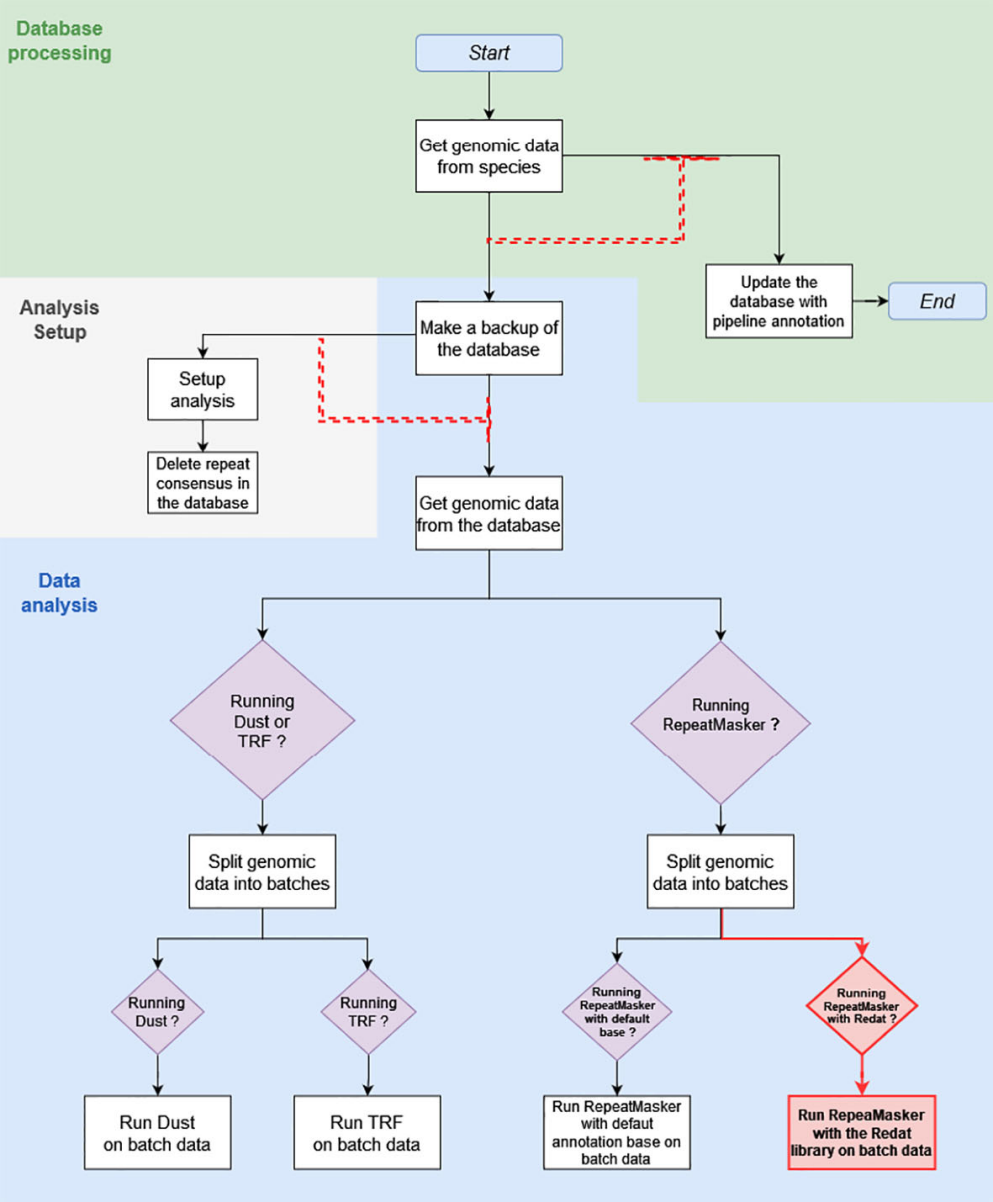
This diagram shows the March 2020 Repeat Features pipeline structure, with modifications made in May 2020 highlighted in red. The white square boxes are the pipeline analyses. Each box is associated with a module, written for the most part in Perl. Boxed purple diamonds are conditional structures. Analyses following these structures are only executed if the condition specified in the diamond is met, in this case when the module specified by the condition has been activated upon pipeline initialization. Black and red arrows show the sequence of analyses. Red dashed arrows are dependent dataflows, where the analysis at the head does not run as long as there are jobs pending in the analysis, or a group of analyses under the arrow base.

What is referred to as the “initial pipeline” is the DNA Features pipeline in its March 2020 version (**Figure 1**). The initial pipeline run used RepeatMasker with default parameters and the Repbase repeat library on all 53 plant genomes of version 39, release 92, of Ensembl. What is referred to as the “updated pipeline” is the DNA Features pipeline in its May 2020 version. A run of the updated pipeline was made with RepeatMasker on low-sensitivity parameters and used REdat as an additional repeat library.

#### 2.3.3 Comparative analysis of repeat elements distributions

**Figure 2:**
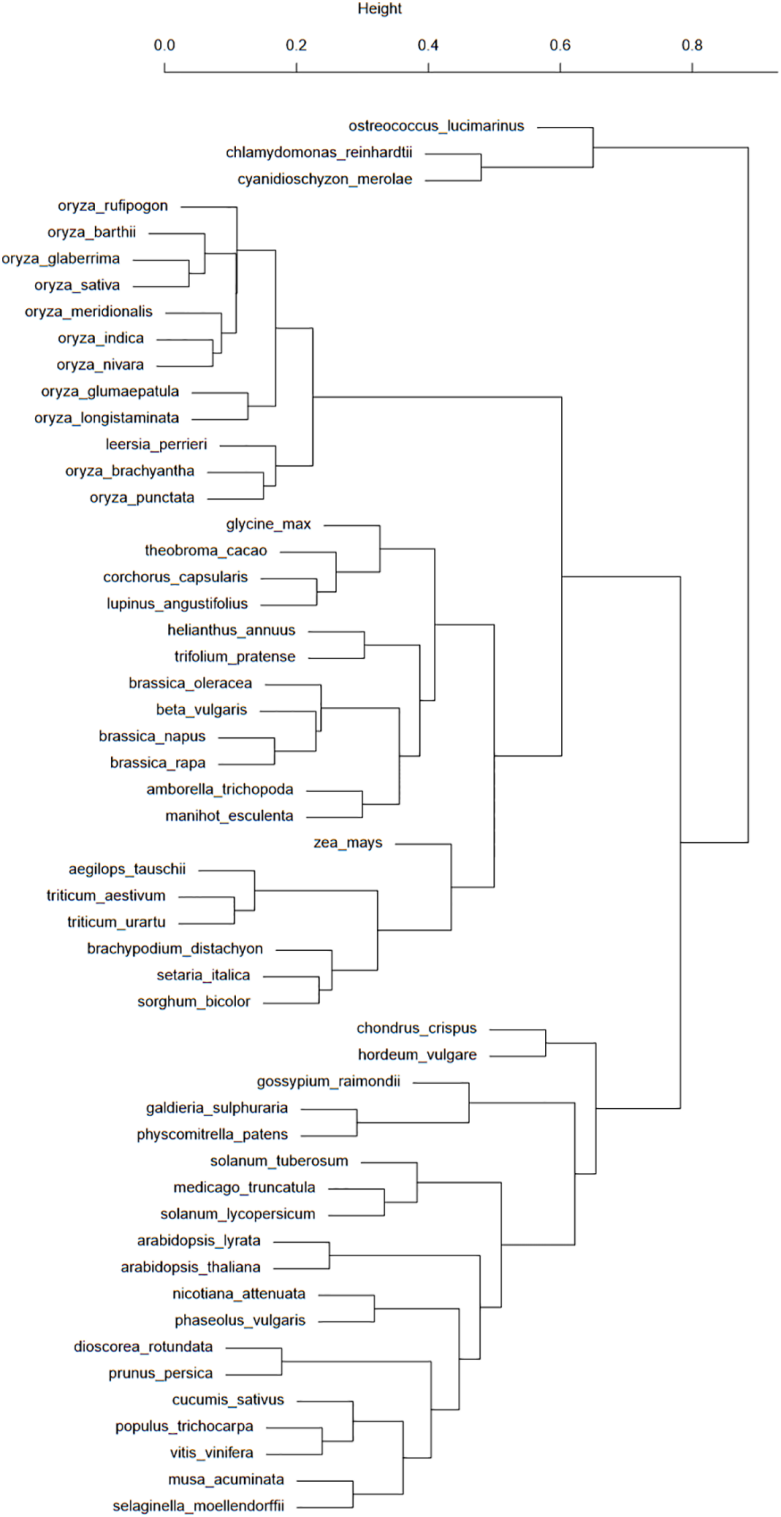
This taxonomic tree was computed from the presence/absence binary matrix of 100 transposons types in the 53 plant species available in the Ensembl database. The top scale shows the relative distances (from 0 to 1) between these species.

**Figure 3:**
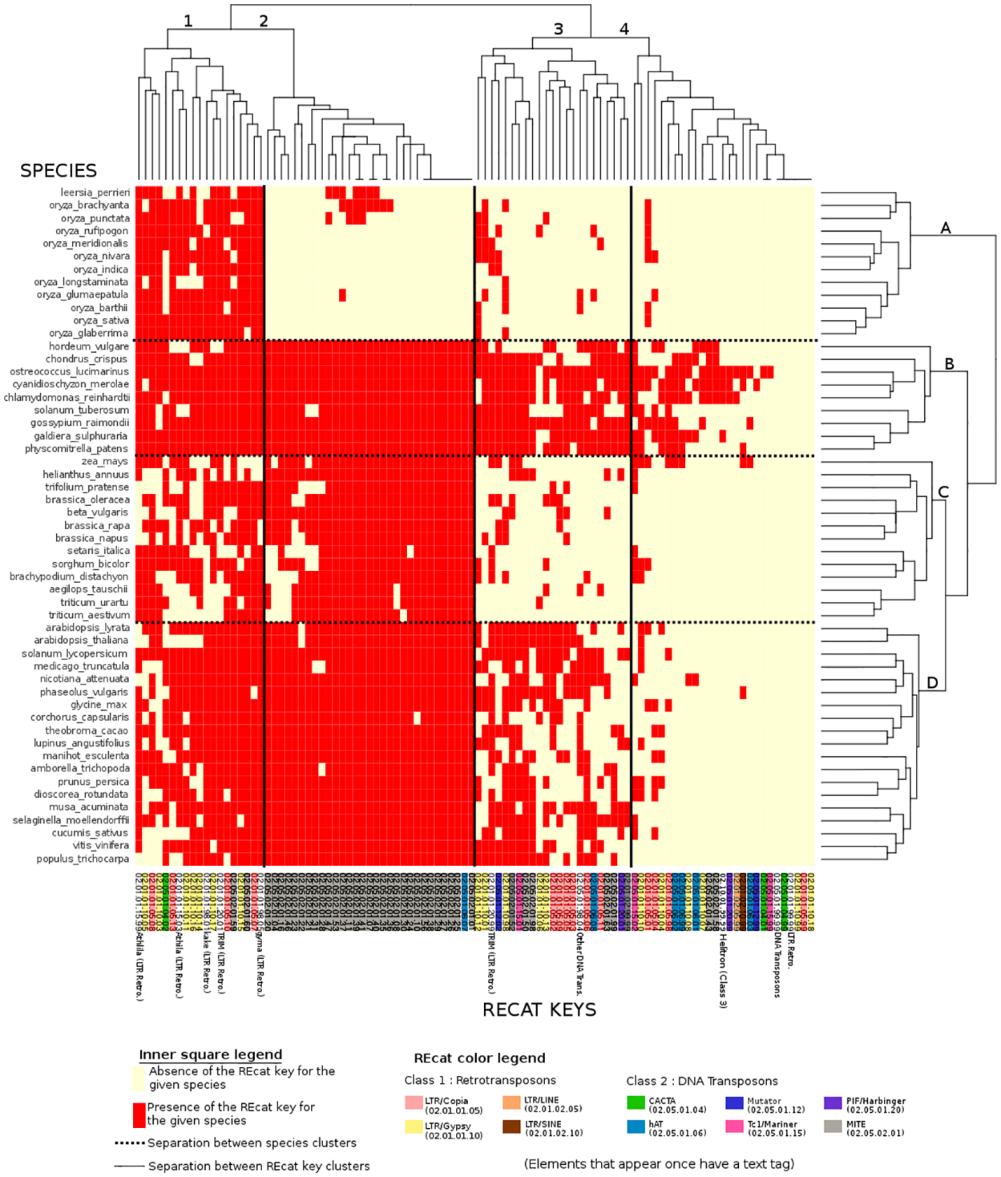
Binary heatmap, where each red point represents the presence of a REcat key on the x-axis in a given species on the y-axis. Each REcat has its five-digit code at the bottom. On the left side is the list of plant species in the Ensembl core database. The top and the left side are trees showing computed relations between keys and between species.

The file containing all repeat element features extracted from the Ensembl database was post-processed by a Perl script to remove every line that was not a transposable element. All REcat keys that had four levels of classification (group, class, type, superfamily, or “unclassified”) were then extended with an additional “unclassified” level, and every REcat key with six levels (group, class, type, superfamily, family, and “unclassified”) was trimmed of their “unclassified” final classification level, using a second Perl script. This modification led to 100 unique REcat keys with five levels. Finally, the processed repeat feature data were treated by a third Perl script. This script used a multi-dimensional hash table as a data structure, with the REcat keys as keys and the species name as value, with this species name also a key for an array of four key metrics, as follows: a binary value for the presence or absence of a key in the given species, number of copies, feature coverage in bp, and feature coverage in the percentage of the genome covered. To compute and visualize the distributions of repeat elements in plant species, all four types of values for every key/species couple were stored in four R vectors and then converted into four matrices of 53 (for 53 species) rows by 100 (for 100 unique REcat keys) columns. The *dist* R module set up distance matrices for the initial 53 by 100 matrices. This module was used with default parameters, except for the “presence/absence” matrix, where the distance parameter used was “binary”, as the values for this particular matrix were binary. Then, the distance matrices were processed with the *hclust* module, also with default parameters, to build clusters from the distance values and then creating views in the form of dendrograms (**Figure 2**). The values of the distance matrices have also been visualized in a heatmap (**Figure 3**).

## 3 Results

### 3.1 Presentation of results in the Ensembl Genome web interface

The data generated from the updated pipeline run have been used as a testing set by the Ensembl Genome team when setting up a web sandbox, and they were made available in the public Ensembl 93 release, with a tag indicating “REdat” data source, to distinguish between Repbase and REdat annotations.

### 3.2 Initial pipeline run and comparison to reported values

To determine if the DNA Features pipeline was sensitive enough to compete or at least come close to current specialized TE detection tools, we compared repeat distributions produced by the pipeline with repeat distributions from the literature that passed through the established quality control.

Total genome coverage and the total number of TE features detected were used as comparison metrics between data sources. The fractions between the pipeline metrics and the article metrics, converted in percentage, were used to determine differences between the pipeline statistics and scientific report statistics (Bolser et al., 2015). On average, the DNA Features pipeline masks 50.32% of reported sequences and detects 116% of reported features. RepeatMasker, with the default library, found too many repetitive structures when compared to what is considered standard. Worse still, this overabundance of detected repetitive structures provides masking that is below this very standard.

**Figure 4:**
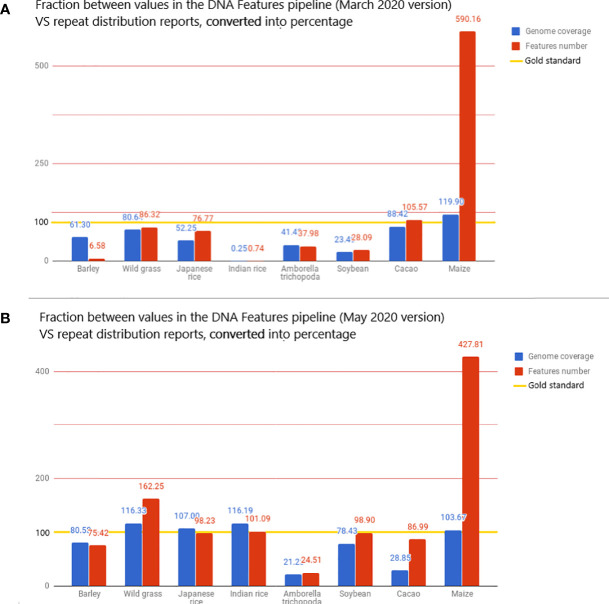
The value displayed is the fraction, converted in percentage, between the metrics from the DNA Features pipeline and repeat distributions from the literature. The reported values are then used here as the gold standard for transposon annotation quality: for each species, the value of genome coverage or the number of features reported in the related article is considered 100% in the bar chart and is highlighted with a gold line. These metrics are the total genome coverage for all transposable elements (blue) and the total number of transposable elements detected (red). The full dataset is available in the Google Docs spreadsheet (Bolser et al., 2015).

The very high value for detected features is mostly due to the extremely high value of features detected for *Z. mays*, as the pipeline detects 490% more features than the literature used as standard. This fivefold increase in the number of reported features results in a genome coverage 20% higher than the standard (**Figure 4A**). When *Z. mays* is removed from the dataset, the pipeline detects, on average, 48.86% fewer features than the reports in the literature. In contrast to the extreme values, the genome coverage and the number of features detected for *O. sativa* sp. *indica* cv. *93-11* are unexpectedly low, with 0.25% of genome masking and 0.74% of feature detection when compared to reports. There is a significant difference in *O. sativa* sp. *indica* between the pipeline statistics and the reference article and also between *O. sativa* sp. *indica* and *O. sativa* sp. *japonica* pipeline statistics. *O. sativa* sp. *japonica* and *O. sativa* sp. *indica*, being cultivars of the same species, should have highly similar repeat distributions.

We suspect that these differences come from the Repbase species-specific annotation, meaning that if few repeats from the Repbase dataset are labeled in the EMBL file as having an “Oryza indica” species annotation, only a few of these repeats are mapped on the *O. sativa* sp. *indica* genome, leading to underestimated statistics. The very high values for *Z. mays* might be the opposite of the same bias, as TEs in *Z. mays* are widely studied. With a plant genome masking 60% under the values considered the golden standard, it has been determined that modifications to the pipeline were relevant. However, these metrics could be restrictive and hide class- or type-specific variations that could only be detected by Repbase. Subsequent updates of Repbase and RepeatMasker could also reduce the significant differences in the considered metrics.

### 3.3 Pipeline extension, test, and rerun

This pipeline extension implemented a new RepeatMasker analysis, similar to the analysis with Repbase (or custom libraries). This new analysis used REdat as a repeat library. Then, the RepeatMasker output, when used with REdat, could be parsed to provide a TE classification.

A new run was performed on the 53 species present in the Ensembl database, with the new analysis. RepeatMasker was used with low sensitivity. The intent was to determine if the implementation showed a significant improvement in the pipeline annotation capabilities. This run using the REdat library increased the average proximity to reference articles by 39% for genome coverage and by 13% for the number of features detected (**Figure 4B**). When compared to the initial pipeline run, the mean genome coverage increased by 22.7% (from 30.16% to 39.02%) and the average number of detected features by 55.36% (from 276,714 to 619,930). This means that running RepeatMasker with REdat on low sensitivity gives better results than RepeatMasker with Repbase on medium sensitivity.

If the extremely low values for Indian rice seem to have been solved, the extremely high values for maize remain after the update. This invalidates the hypothesis of the species-specific system in Repbase and raises a new hypothesis: it could be due to the variation in the number of reported transposable elements in a given species.

The high number of species that have values superior to 100% raises the question of the specificity behind the sensitivity or the number of false positives in the updated run and the need for manual validation. It could also benefit in the long run with higher RepeatMasker sensitivity.

### 3.4 Comparison between species in the context of the known taxonomy

Two figures, a dendrogram (**Figure 2**) and a heatmap (**Figure 3**), were produced from the comparative analysis of repeat distributions, using the presence/absence metric. Analysis heatmaps and dendrograms were produced for the three other metrics (copy number, feature coverage in bp, and percentage of the genome covered) but did not show significant results.

**Figure 2** shows a good classification of rice and grasses in a common branch. However, the fact that this common branch is also populated with a large group of eudicots raises some questions about the TE history of these elements. One particular case of this separation of eudicots is about the Brassicaceae, with the *Brassica* genus in the branch comprising monocots and eudicots and the *Arabidopsis* genus in the “eudicots-only” branch. These species are separated by many events of whole genome duplications (Chalhoub et al., 2014). This study asks questions about the impact of whole genome duplications on transposon distribution and activity. Another case worth investigating is the presence of *H. vulgare* and *Gossypium raimondii* among algae and mosses.

If the dendrogram bootstrap has not been performed, its strength can nonetheless be assessed with the clusters from **Figure 2**. As grasses are grouped with eudicots, this branch position could be considered unstable. *H. vulgare* is still grouped with mosses and algae, allowing us to reiterate our questions about barley TE history.

In the binary heatmap, the LTR/copia and LTR/gypsy superfamilies are spread over clusters 1, 2, and 4. Moreover, it is worth noting that cluster 4, which represents the most keys distributed around species, is mainly composed of LTR, which can be explained by the retroviral origin of these elements (Hayward, 2017). Cluster 4 also has two types of miniature inverted-repeat transposable elements (MITEs) that are known to have a large history of horizontal transfers (Zhang et al., 2018). However, the presence of a DNA Transposon/CACTA superfamily in this cluster is left unexplained.

MITEs, DNA transposons in general, are mostly absent from species cluster A. However, they are present in eudicots, algae, mosses, and other monocots, raising questions about the genetic appearance or removal event that occurred with MITEs and DNA transposons in rice.

Finally, this analysis is based on a binary matrix, and it could benefit from a deeper analysis using non-binary values. Moreover, the REcat key system has been altered to overcome Perl limitations. If this alteration still provides a solid analysis, with a hundred keys taken into account, an analysis using an imposed hierarchy tree and every REcat key available could provide more precise information.

## 4 Conclusions

The high number of repeat elements in plant genomes was a significant challenge in Ensembl’s quest to annotate and align genomes. The detection of these elements by the DNA Features pipeline also had phylogenetic implications in the determination of repeat expansions and their subsequent removals. However, using RepeatMasker with Repbase, a library of eukaryotes, showed limitations. The implementation of the REdat repeat library proved to be needed and efficient, compared to repeat distribution from reference scientific articles. The new classification associated with REdat, REcat, also allowed a comparative analysis of the repeat element distributions in the 53 species available in the Ensembl Genome in 2020. The dendrogram from this comparative analysis showed promising results (**Figure 2**), in particular with monocots. However, strong discrepancies with the expectations, especially with *H. vulgare*, or the Brassicaceae, need to be investigated. The heatmap associated with this analysis shows the absence of MITEs in most species of rice, the presence of LTRs in every species cluster, and DNA transposons in a cluster comprising mosses, algae, “outliers”, and *H. vulgare*. These particular clusterings need to be investigated, in addition to the differences between taxonomic space and repeat distributions.

## Data availability statement

The datasets presented in this study can be found in online repositories. The names of the repository/repositories and accession number(s) can be found below: https://docs.google.com/spreadsheets/d/1kMMckERzqy9gwsFVWELfuj9q0dSKxh5IXL1D_S9wOug/edit#gid=359252355.

## Author contributions

Bioinformatics execution, figure rendering, code editing and writing was made by JA. DB was Ensembl Plants team leader, JA’s intership supervisor and provided scientific input, advise and data. PK and PF were respectively leaders of the Ensembl non-vertebrate genomics and vertebrate genomics teams. All authors contributed to the article and approved the submitted version.

## Footnotes

1 Smit, AFA, Hubley, R & Green, P. RepeatMasker Open-4.0. 2013-2015 http://www.repeatmasker.org

2 IBM’s LSF product page, https://www.ibm.com/products/hpc-workload-management

3 eHive production system page on Ensembl documentation, https://www.ensembl.org/info/docs/eHive.html
